# Comparing the performance of QuantiFERON-TB Gold Plus with QuantiFERON-TB Gold in-tube among highly TB exposed gold miners in South Africa

**DOI:** 10.12688/gatesopenres.13191.1

**Published:** 2021-03-11

**Authors:** Thobani Ntshiqa, Violet Chihota, Raoul Mansukhani, Lindiwe Nhlangulela, Kavindhran Velen, Salome Charalambous, Pholo Maenetje, Thomas R. Hawn, Robert Wallis, Alison D. Grant, Katherine Fielding, Gavin Churchyard

**Affiliations:** 1 Implementation Research Division, The Aurum Institute, Johannesburg, Gauteng, 2193, South Africa; 2 School of Public Health, University of the Witwatersrand, Johannesburg, Gauteng, 2193, South Africa; 3 Department of Infectious Disease Epidemiology, London School of Hygiene & Tropical Medicine, London, United Kingdom, WC1E 7HT, UK; 4 Department of Medicine, University of Washington, Seattle, Seattle, New York, 98195, USA; 5 Africa Health Research Institute, Laboratory Medicine & Medical Sciences, College of Health Sciences, University of KwaZulu-Natal, Durban, KwaZulu-Natal, 4041, South Africa

**Keywords:** Latent Tuberculosis Infection, QFT-GIT, QFT-Plus, TST, Performance, Goldmines, South Africa

## Abstract

**Background: **QuantiFERON-TB Gold in-tube (QFT-GIT) is an interferon-gamma release assay (IGRA) used to diagnose latent tuberculosis infection. Limited data exists on performance of QuantiFERON-TB Gold-Plus (QFT-Plus), a next generation of IGRA that includes an additional antigen tube 2 (TB2) while excluding TB7.7 from antigen tube 1 (TB1), to measure TB specific CD4+ and CD8+ T lymphocytes responses. We compared the performance of QFT-Plus with QFT-GIT among highly TB exposed goldminers in South Africa.

**Methods:** We enrolled HIV-negative goldminers in South Africa, ≥33 years with no prior history of TB disease or evidence of silicosis. Blood samples were collected for QFT-GIT and QFT-Plus. QFT-GIT was considered positive if TB1 tested positive; while QFT-Plus was positive if both or either TB1 or TB2 tested positive, as per manufacturer's recommendations. We compared the performance of QFT-Plus with QFT-GIT using Cohen’s Kappa. To assess the specific contribution of CD8+ T-cells, we used TB2−TB1 differential values as an indirect estimate. A cut-off value was set at 0.6. Logistic regression was used to identify factors associated with having TB2-TB1>0.6 difference on QFT-Plus.

**Results:** Of 349 enrolled participants, 304 had QFT-Plus and QFT-GIT results: 205 (68%) were positive on both assays; 83 (27%) were negative on both assays while 16 (5%) had discordant results. Overall, there was 94.7% (288/304) agreement between QFT-Plus and QFT-GIT (Kappa = 0.87). 214 had positive QFT-Plus result, of whom 202 [94.4%, median interquartile range (IQR): 3.06 (1.31, 7.00)] were positive on TB1 and 205 [95.8%, median (IQR): 3.25 (1.53, 8.02)] were positive on TB2. A TB2-TB1>0.6 difference was observed in 16.4% (35/214), with some evidence of a difference by BMI; 14.9% (7/47), 9.8% (9/92) and 25.3% (19/75) for BMI of 18.5-24.9, 18.5-25 and >30 kg/m ^2^, respectively (P=0.03).

**Conclusion: **In a population of HIV-negative goldminers, QFT-Plus showed a similar performance to QFT-GIT.

## Introduction

Latent tuberculosis infection (LTBI) is the seedbed from which tuberculosis (TB) cases arise. LTBI is defined as an asymptomatic state characterized with a persistent immune response to stimulation by *Mycobacterium tuberculosis* (Mtb) antigens with no evidence of active TB ^1– 3^. LTBI is typically characterized by a positive tuberculin skin test (TST) *in vivo*, involving intradermal injection of purified protein derivative from Mtb strain and/or a positive interferon-gamma release assay (IGRA) ^4, 5^. Better tests are needed to identify persons at increased risk of developing TB disease.

IGRAs measure released interferon-gamma from cluster of differentiation (CD) T-lymphocytes specific to Mtb complex antigens but not produced by *Mycobacterium bovis* BCG vaccine strains ^6^. QuantiFERON-TB Gold In-Tube assay (QFT-GIT) is designed to elicit interferon-gamma response from CD4+ helper T lymphocytes in a single TB antigen tube containing long peptides from ESAT-6, CPF-10 and TB7.7 antigens (Qiagen, Germantown, MD) ^5– 7^. QuantiFERON-TB Gold Plus assay (QFT-Plus) is a next generation IGRA that contains peptides from only the ESAT-6 and CFP-10 antigens comprising a TB1 tube, identical to the QFT-GIT, with the exception of TB7.7, and stimulates CD4+ T cells, and an additional antigen tube, TB2, which has a cocktail of both long and short ESAT-6 and CFP-10 peptides to elicit interferon-gamma release from both CD4+ helper T lymphocytes and CD8+ cytotoxic T lymphocytes ^5– 8^.

CD8+ cytotoxic T lymphocytes stimulating peptide was included in the QFT-Plus assay to improve on the sensitivity of QFT-GIT. Currently, limited data exist comparing the performance of these assays in high TB burdened settings. We compared the performance of QFT-Plus with QFT-GIT among highly Mtb exposed goldminers in South Africa.

## Methods

### Study setting and population

The study was conducted in the South African goldmines at the Occupational Health Centre (OHC), in Orkney, North West Province among goldminers, attending for their annual medical examination between July 2015 and December 2016.

### Study design and procedures – parent study

In a cohort study, herein described as the parent study, we enrolled miners to identify those who were uninfected with Mtb despite being highly exposed to Mtb, to compare epidemiological factors between Mtb uninfected and infected miners and to collect specimens from Mtb uninfected and infected miners to determine gene expression and immunological profiles associated with being Mtb uninfected in future analysis.

Goldminers, attending OHC for their annual medical examination were pre-screened for the study to identify those aged 33-60 years who had worked in the mining industry for at least 15 years. Following informed consent, a full screen was conducted. Miners were included if they did not have symptoms suggestive of TB, no prior or current history of treatment for active TB disease, no history of or not currently taking isoniazid preventive therapy, no silicosis, had body mass index (BMI) >18.5, no serious medical conditions, HIV negative and no current treatment for cancer, no treatment with steroid tablets, inhalers or injections.

Blood samples were collected intravenously by trained professional phlebotomy nurses amongst those who met the inclusion criteria for QFT (QFT-Plus and QFT-GIT; Qiagen, Hilden Germany), peripheral blood mononuclear cell (PBMC), and transcriptomic (PAXgene) testing. Under the first version of the protocol (enrolments from 10 July 2015 to 29 October 2015), participants gave blood samples for QFT at enrolment and were followed up at 90 days where QFT was repeated and TST (RT-23 in Tween-80, Statens Serum Institute, Copenhagen, Denmark, catalogue numbers: XI1177KL and SP18411C) was placed and read by a professional nurses who were trained in placing and reading TST. The protocol was subsequently simplified and under the second version (enrolments after 29 October 2015) participants gave blood samples for QFT at enrolment and TST placed either at enrolment or seven days later. All participants, regardless of protocol version, were followed up to 12 months where QFT and TST were repeated.

A sputum sample was also collected for mycobacterial culture testing (BACTEC MGIT 960 system, BD Diagnostic Systems, Sparks, MD, USA) to exclude subclinical TB. A questionnaire was administered in a private room by a trained research assistant to collect demographic characteristics and information on factors associated with being TB uninfected. Blood samples collected were tested at the Aurum Clinical Research laboratory for LTBI using QFT-GIT and QFT-Plus. A 6 ml of whole blood sample was collected intravenously from each participant into a single lithium heparin tube. Samples were then transported to Aurum Clinical Research laboratory where they were aliquoted into 1 ml tubes: three tubes for QFT-GIT test kit (QFT-GIT nil, QFT-GIT TB, QFT-GIT mitogen with catalogue numbers 0594-0201 and 0594-0501) and four for QFT-Plus (QFT-Plus nil, QFT-Plus TB1, QFT-Plus TB2, QFT-Plus mitogen with catalogue number 622120). Tubes were then placed in a pre-heated 37°C portable incubator for 16-24 hours, within eight hours of collection. Subsequently, samples were centrifuged to separate plasma for same day testing. Alternatively, samples were stored at -80°C for up to 48-72 hours prior testing. Plasma was tested by interferon-gamma enzyme-linked immunosorbent assay (ELISA), performed using Biotek microplate reader model EL x 800 using Gen 5 software. Biotek microplate washer model EL x 508 and Thermostar shaker were also used.

### Study design – sub-study

In this sub-study, using cross-sectional data from the parent study, we compared the performance of QFT-Plus with QFT-GIT using QFT and TST measurements all done either at baseline/seven days (participants enrolled under the 2 ^nd^ version of protocol) or at 90 days post-enrolment (participants enrolled under the 1 ^st^ version of protocol).

### Study definitions

QFT-GIT was considered positive if TB1 tested positive; while QFT-Plus was positive if both TB1 and TB2 tested positive or if either TB1 or TB2 tested positive, as per manufacturer's recommendations. TST induration ≥5mm was considered positive. To assess the specific contribution of CD8+ T-cells, we used TB2−TB1 differential values as an indirect estimate. A cut-off value was set at 0.6 in order to reduce the bias of the intrinsic variability of the test ^9^.

### Statistical methods

All eligible participants from the parent study were included in the analysis. Binary outcomes (positive/negative) from QFT-Plus and QFT-GIT were compared using the percentage agreement and Kappa statistic. TB1 and TB2 responses for those QFT-Plus positive were summarized by QFT-GIT status using median and interquartile range (IQR) and percentage positive (measurement minus nil response>0.35). TST positivity was summarized by QFT-Plus status. Logistic regression was used to identify factors associated with having TB2-TB1 >0.6. Results were summarized using odds ratios (OR) with their corresponding 95% confidence intervals (CI) and p-values. Due to a small number of outcomes a multivariable analysis was not conducted. Data were analyzed using Stata version 15 (StataCorp. 2017. Stata Statistical Software: Release 15. College Station, TX: StataCorp LP.

### Ethical statement

The study received ethical clearance from the University of Witwatersrand Human Research Ethics Committee (WHREC Ref: 150217), London School of Hygiene & Tropical Medicine, UK (LSHTM Ethics Ref: 9279), University of Washington, USA (IRB number 33335) and North West Health Research and Ethics Committee (DOH-27-0515-4991). We sought informed consent from all study participants using written informed consent and information sheets available in the most commonly used local languages. Participants who were unable to read or write were asked to make a mark or thumbprint in the presence of a witness. This study was conducted according to Good Clinical Practice guidelines, in accordance with the requirements of the funders and respective ethics committees.

## Results

### Process flow

**Figure 1.**
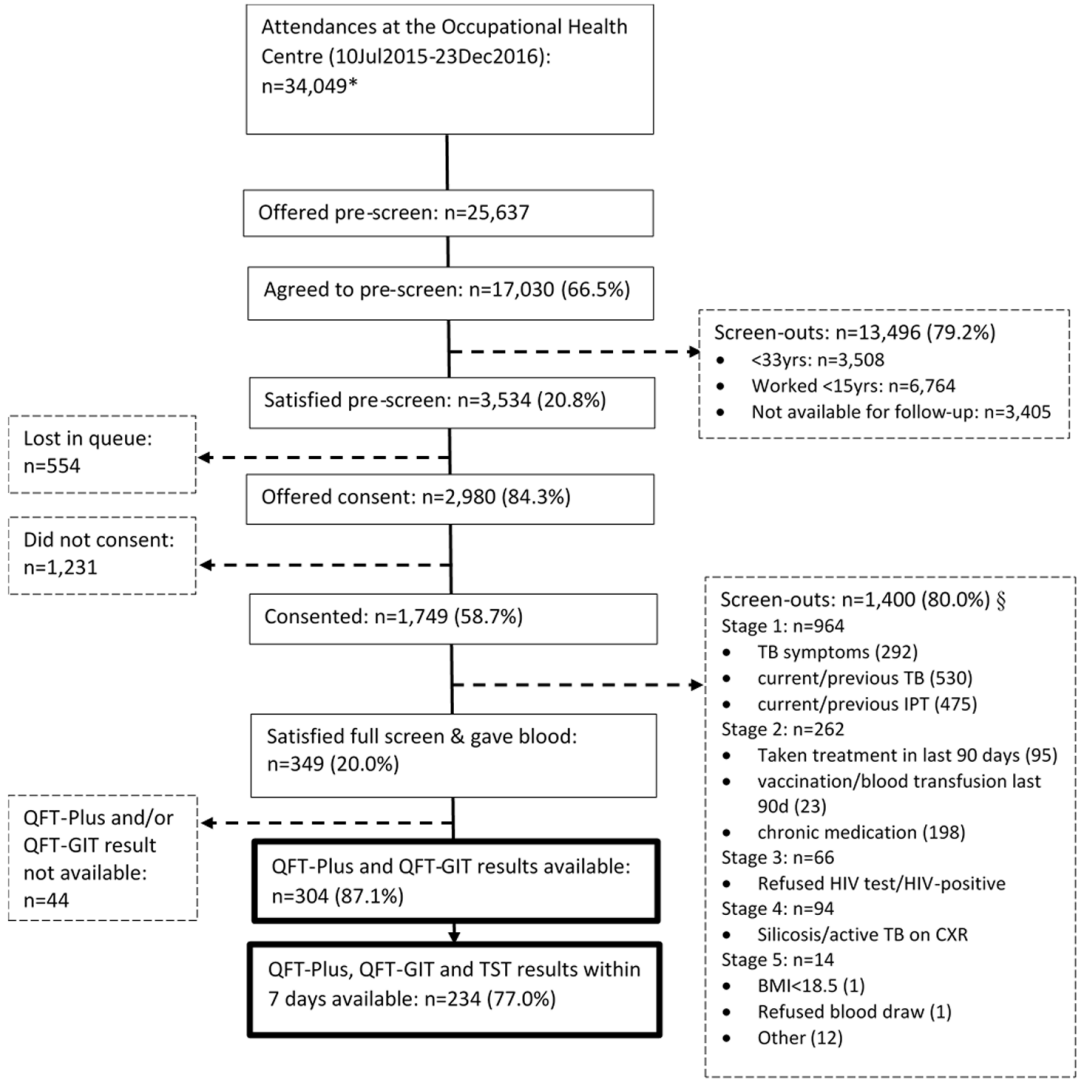
Participant flow chart at enrolment. *Data from the daily attendance register. Includes repeat attendances during this time period. §Screen out questions were asked in stages; if screened out at a stage no screening was conducted for subsequent stages. Within a stage more than one screen-out reason may apply. Yrs, years; IPT, isoniazid preventive therapy; BMI, body mass index; QFT-Plus, QuantiFERON-TB Gold-Plus; QFT-GIT, QuantiFERON-TB Gold In-Tube assay; TST, tuberculin skin test.

We approached 25,627 miners, 17,030 (66.5%) agreed to be pre-screened, of whom 3,534 (20.8%) satisfied the pre-screen criteria and were eligible for full screening, following informed consent ( Figure 1). Overall, 2,980 (84.3%) were offered consent, of whom 1,749 (58.7%) consented and 1,231 (41.3%) declined to take part in the study; 554 (15.7%) were not offered consent as they were lost in the OHC queue.

Following the full screen, 349/1749 (20.0%) met the inclusion criteria and were enrolled into the study. Of the 349, 304 had a baseline QFT-Plus and QFT-GIT while 234 had QFT-GIT, QFT-Plus and TST measured within seven days of each other. For the latter, which is restricted to having a TST measurement within seven days of the QFT measurements, participants enrolled under the 1st version of the protocol contribute QFT-Plus and QFT-GIT measurements from their 90-day visit.

### Demographic characteristics

Of the 349 participants enrolled into the study, the median age was 48 years (IQR 45, 53 years), median years in the workface was 24 (IQR 18, 28 years) and 98.6% (344) were male ( Table 1). Overall, 92.0% (321) were of Black/African ethnicity, 72.5% (253) had a BCG scar present, 66.8% (233) were born in South Africa and a minority lived in a mine hostel (28.7%; 100). Subsamples of the 349 participants who either had (i) a baseline QFT-Plus and QFT-GIT result (n=304) or (ii) QFT-Plus and TST reading (n=234) had similar demographic characteristics to the overall sample ^10^.

**Table 1.** Summary of demographic characteristics at enrolment.

Variable		Participants Enrolled	Participants enrolled with a QFT-Plus and QFT-GIT result	Participants enrolled with a QFT-Plus, QFT-GIT & TST result
	N	349	304	234
**Age, years**	Median (IQR)	48 (45-52)	48 (44-52)	48.5 (44-52)
**Sex**	Male	344 (98.6%)	300 (98.7%)	230 (98.3%)
**BCG scar**	No	84 (24.1%)	74 (24.3%)	58 (24.8%)
	Yes	253 (72.5%)	221 (72.7%)	171 (73.1%)
	Indeterminate	12 (3.4%)	9 (3.0%)	5 (2.1%)
**Country of birth**	South Africa	233 (66.8%)	204 (67.1%)	157 (67.1%)
	Lesotho	59 (16.9%)	50 (16.4%)	38 (16.2%)
	Mozambique	37 (10.6%)	32 (10.5%)	26 (11.1%)
	Other	20 (5.7%)	18 (5.9%)	13 (5.6%)
**Ethnicity**	Black/African	321 (92.0%)	279 (91.8%)	218 (93.2%)
**Hostel**	No	170 (48.7%)	155 (51.0%)	122 (52.1%)
	Hostel	100 (28.7%)	83 (27.3%)	58 (24.8%)
	Other mine house	79 (22.6%)	66 (21.7%)	54 (23.1%)
**Years worked** **underground**	Median (IQR)	24 (18-28)	24 (17.5-28)	24 (17-28)
**Sleeping** **arrangement**	Alone	51 (14.6%)	46 (15.1%)	34 (14.5%)
	1 person	206 (59.0%)	182 (59.9%)	139 (59.4%)
	> 1 person	92 (26.4%)	76 (25.0%)	61 (26.1%)
**Occupation**	Unskilled	271 (77.7%)	236 (77.6%)	188 (80.3%)

IQR, interquartile range; QFT-Plus, QuantiFERON-TB Gold-Plus; QFT-GIT, QuantiFERON-TB Gold; TST, tuberculin skin test.

### Comparison of QuantiFERON-TB Gold-Plus vs. Gold in-tube

Of the 304 who had QFT-Plus and QFT-GIT results, 214 (70.4%) had a positive QFT-Plus result, 205 (67.4%) were positive on both assays; 83 (27.3%) were negative on both assays, while 16 (5.3%) had discordant results (seven QFT-Plus negative/QFT-GIT positive; nine QFT-Plus positive/QFT-GIT negative) ( Table 2). Overall, the agreement between QFT-Plus and QFT-GIT was 94.7% (288/304) and Kappa was 0.87.

**Table 2.** Comparison of QuantiFERON-TB Gold-Plus vs. QuantiFERON-TB Gold in-tube.

Participants who have QFT-Plus and QFT-GIT, TST data not shown									
QFT-GIT	N	QFT-Plus		QFT-Plus interferon-gamma concentration, among those positive on QFT- Plus					
		Positive	Negative	Positive result *: TB1 (%)	Positive result *:TB2 (%)	TB1-nil Median (IQR)	TB2-nil Median (IQR)	TB2 - TB1 median difference (IQR)	TB2 - TB1 >0.6
Positive	212	205	7	198/205 ^a^ (96.6%)	200/205 ^b^ (97.6%)	3.06 (1.31, 7)	3.25 (1.53, 8.02)	0.00 (-0.16,0.39)	34/205 (16.6%)
Negative	92	9	83	4/9 ^c^ (44.4%)	5/9 ^d^ (55.6%)	0.35 (0.18, 0.53)	0.37 (0.28, 0.45)	0.02 (-0.23,0.30)	1/9 (11.1%)
Total	304 ^	214	90	202/214 (94.4%)	205/214 (95.8%)	2.89 (1.18, 6.97)	2.95 (1.17, 7.79)	0.00 (-0.17,0.39)	35/214 (16.4%)
Participants who have QFT-Plus, QFT-GIT and a TST measurement within seven days of the QFT results									
QFT-GIT	N	QFT-Plus		QFT-Plus interferon-gamma concentration, among those positive on QFT- Plus					
		Positive	Negative	Positive result *: TB1 (%)	Positive result *:TB2 (%)	TB1-nil Median (IQR)	TB2-nil Median (IQR)	TB2 - TB1 median difference (IQR)	TB2 - TB1 >0.6
Positive	166	160 ^1^	6 ^2^	154 ^e^ /160 96.2%	157 ^f^/160 98.1%	2.91 (1.22, 6.97)	2.88 (1.37, 7.57)	0 (-0.17,0.39)	26/160 16.25%
Negative	68	6 ^3^	62 ^4^	3/6 ^g^ 50%	3/6 ^h^ 50%	0.34 (0.18, 0.53)	0.36 (0.28, 0.45)	0.02 (-0.23, 0.3)	0/6 0%
Total	234 ^^	166	68	157/166 94.6%	160/166 96.4%	2.70 (1.07, 6.88)	2.74 (1.13, 7.25)	0 (-0.17, 0.38)	26/166 15.7%

^ n=304 who have QFT-Plus and QFT-GIT. Percentage agreement 94.7% (95%CI: 91.6– 97.0%); Kappa 0.87. ^^ n=234/304 who have QFT-Plus and QFT-GIT and a TST measurement within seven days of the QFT results. For participants enrolled under the 1 ^st^ version of the protocol the QFT-Plus and QFT-GIT measurements are from the 90-day visit. Percentage agreement 94.9% (95%CI: 91.2 – 97.3%); Kappa 0.88. ^1 ^61.3% [98/160] TST positive (defined as ≥5mm); ^2^ 33.3% [2/6] TST positive; ^3^ 50.0% [3/6] TST positive; ^4^ 40.3% [25/62] TST positive. ^a^ Of the 198, n=5 are positive on TB1 alone; ^b^ of the 200, n=7 are positive on TB2 alone; ^c^ of the four, all are positive on TB1 alone; ^d^ of the five, all are positive on TB2 alone. ^e^ Of the 154, n=3 are positive on TB1 alone; ^f^ of the 157, n=6 are positive on TB2 alone; ^g^ of the three, all are positive on TB1 alone; ^h^ of the three, all are positive on TB2 alone. * Defined as TB1-nil>0.35 or TB2-nil>0.35. QFT-Plus, QuantiFERON-TB Gold-Plus; QFT-GIT, QuantiFERON-TB Gold in-tube; IQR interquartile range; CI confidence interval.

Two hundred and thirty-four participants had QFT-Plus, QFT-GIT and TST measured at the same time/or within seven days of each other. Overall, 54.7% (128) had a TST response ≥5mm, 11.1% (26) had a TST response >0mm and <5mm and 34.2% (80) had zero TST response. The median TST response was 11mm (IQR 6-15mm), among those with a TST>0mm. Of the 234, 160 (68.4%) were positive on both assays, of whom 61.3% (98/160) were TST-positive, and 62 (26.5%) were negative on both assays, of whom 40.3% (25/62) TST-positive. Furthermore, 101 (43.2%) were QFT-Plus positive/TST positive; 41 (17.5%) were QFT-Plus negative/TST negative; 65 (27.8%) were QFT-Plus positive/TST negative; and 27 (11.5%) were QFT-Plus negative/TST positive. However, 12 (5.1%) had discordant results [six QFT-GIT positive/QFT-Plus negative, 33.3% (2/6) TST positive; six QFT-GIT negative/QFT-Plus positive, 50.0% (3/6) TST positive]. When analysis was restricted to a subsample of 234, the percentage agreement between QFT-GIT and QFT-Plus was 94.9%, similar to before.

### Assessing the specific contribution of CD8+ T-cells

Of the 214 who had positive QFT-Plus results, 202 (94.4%) were positive on TB1, while 205 (95.8%) were positive on TB2 ( Table 2). The median (IQR) values for TB1 and TB2 among those who were QFT-Plus positive were 3.06 (1.31, 7.00) and 3.25 (1.53, 8.02), respectively. The median difference between the TB1 and TB2 was 0.00 (-0.17, 0.39). A >0.6 difference between TB2 and TB1 was observed in 16.4% (35/214) of those who were positive on QFT-Plus ( Table 2). Only BMI status was found to be associated with TB2-TB1 >0.6; unadjusted OR 1.94 (95% CI: 0.74–5.05) for BMI>30 kg/m ^2^ versus BMI between 18.5-24.9 ( Table 3).

**Table 3.** Risk factors for having TB2-TB1>0.6 on QFT-plus.

Variable	N	TB2-TB1 >0.6 n (%)	Univariable analysis		
			Crude OR	95% CI	p-value *
**Age group, years**					0.85
<45	48	7 (14.6)	1		
45–49	71	13 (18.3)	1.31	0.48 - 3.58	
≥50	95	15 (15.8)	1.10	0.42 - 2.91	
**Gender**					0.66
Male	210	34 (16.2)	1		
Female	4	1 (25.0)	1.73	0.17 - 17.09	
**Ethnicity**					0.20
Black/African	209	33 (15.8)	1		
Other	5	2 (40.0)	3.56	0.57 - 22.11	
**Marital status**					0.14
Married	193	29 (15.0)	1		
Other	21	6 (28.6)	2.26	0.81 - 6.31	
**Country of birth**					0.85
South Africa	141	25 (17.7)	1		
Lesotho	39	5 (12.8)	0.68	0.24 - 1.92	
Mozambique	23	3 (13.0)	0.70	0.19 - 2.52	
Other	11	2 (18.2)	1.03	0.21 - 5.07	
**Occupational level**					0.17
Unskilled	177	26 (14.7)	1		
Skilled	37	9 (24.3)	1.87	0.79-4.41	
**Years worked underground**					0.99
<20	61	10 (16.4)	1		
20-29	112	18 (16.1)	0.98	0.42 - 2.27	
≥30	41	7 (17.1)	1.05	0.36 - 3.03	
**Type of mine housing**					0.31
Not staying in mine house	102	17 (16.7)	1		
Hostel	68	8 (11.8)	0.67	0.27 - 1.64	
Other mine housing	44	10 (22.7)	1.47	0.61 - 3.53	
**Sleeping arrangement**					0.30
Alone	38	5 (13.2)	1		
1 person	129	25 (19.4)	1.59	0.56 - 4.48	
>1 person	47	5 (10.6)	0.79	0.21 - 2.94	
**BCG Scar**					0.39
Yes/indeterminate ^¥^	159	28 (17.6)	1		
No	55	7 (12.7)	0.68	0.28-1.66	
**Transverse diameter of TST** induration, mm **					0.33
<5	65	8 (12.3)	1		
≥5	101	18 (17.8)	1.55	0.63 - 3.79	
**BMI, kg/m ^2^**					0.03
18.5-24.9	47	7 (14.9)	1		
25-29.9	92	9 (9.8)	0.62	0.22 - 1.78	
≥30	75	19 (25.3)	1.94	0.74 - 5.05	

* P-value from the likelihood ratio test; ** n=166; ¥, n=4 indeterminate. QFT-Plus, QuantiFERON-TB Gold-Plus; TST, tuberculin skin test; OR, odds ratio; CI confidence interval.

## Discussion

In this study, conducted in South African goldmines, the overall agreement between QFT-Plus and QFT-GIT was high at 94.7%, suggesting that QFT-GIT may have similar performance to QFT-Plus; consistent with previous evaluation studies conducted in low TB incidence settings which showed similar diagnostic performance and high overall agreement between QFT-Plus and its predecessor ^5, 8, 11– 15^. The high concordance (or low discordance) in our study may be because our study was in HIV negative adults among whom QFT-GIT would be expected to have relatively high sensitivity. A greater difference between the two tests might be expected in populations where QFT-GIT typically has poor sensitivity e.g. children and people with advanced HIV disease ^11, 16– 18^. Overall, there were 16 discordant pairs (5.3%); seven were QFT-Plus negative/QFT-GIT positive and nine were QFT-Plus positive/QFT-GIT negative. It is interesting that only 56.3% were QFT-Plus positive/QFT-GIT negative and not higher. The discordancy rate found in our study was consistent with findings from Theel *et al.* and Moon *et al*. studies, which showed discordancy rates of 3.1% and 4.4%, respectively ^5, 12^. However, of the five discordant pairs in the Theel *et al.* study, 60.0% (3/5) were QFT-Plus negative/QFT-GIT positive and 40.0% (2/5) were QFT-Plus positive/QFT-GIT negative; while in the Moon *et al.* study, 25.6% (11/43) were QFT-Plus negative/QFT-GIT positive and 74.4% (32/43) were QFT-Plus positive/QFT-GIT negative in the 43 discordant pairs. This is thought to be due to several factors, broadly classified as preanalytical, analytical, postanalytical, manufacturing, immunological, and interferon-gamma levels bordering on the binary 0.35 IU/ml cutoff for assay positivity ^9, 19– 21^.

We observed a TB2−TB1 difference >0.6 among 35 (16.4%) individuals who had QFT-Plus positive results, associated with obesity. Barcellini *et al.* had also observed a similar TB2−TB1 difference >0.6 in a small proportion of TB contacts who had a positive QFT-Plus results 18 (15.1%); suggesting presence of Mtb-specific CD8+ T lymphocytes, which may be indicative of a higher antigenic burden ^14, 22– 27^. The presence of Mtb-specific CD8+ T lymphocytes in latently infected miners may therefore be predictive of Mtb active replication and may be indicative of higher likelihood of disease progression ^23^.

### Study limitations

This sub-analysis was based on a cross-sectional sample and did not include follow-up to confirm the LTBI status. However, in this population of goldminers with long duration of service, equating to prolonged exposure to Mtb infection, it is unlikely that the LTBI status would change over a relatively short period of follow-up. The sample size of 304 was relatively small to make strong inferences. In addition, the study was conducted among HIV-negative goldminers, selected for those most likely to have experienced Mtb exposure, thus the results may not be generalizable. The lack of a gold-standard test for Mtb infection means that where results were discordant, we cannot know which (if either) was correct.

## Conclusion

Among HIV-negative goldminers in South Africa, the performance of QFT-Plus was similar to QFT-GIT. For most discordant results, interferon-gamma concentrations bordered on the binary cutoff for assay positivity.

## Data availability

LSHTM Data Compass: Data set for the comparison of the performance of QuantiFERON-TB Gold Plus with QuantiFERON-T Gold in-tube among highly TB exposed gold miners in South Africa. https://doi.org/10.17037/DATA.00001891 ^10^.

This project contains the following underlying data:

- Hetu-dataset.txt
- Hetu_data_codebook.html
- Hetu_data_userguide.html

Due to ethical concerns, dataset access is restricted to ensure privacy and confidentiality of participant data. However, raw data is available upon request under a custom data sharing agreement and will require authorization from Principal Investigators (Professor Violet Chihota: VChihota@auruminstitute.org and Professor Katherine Fielding: Katherine.Fielding@lshtm.ac.uk). Once access is granted, the files will be made available on LSHTM Data Compass.

The data codebook and user guide are available under the terms of the Creative Commons Attribution 3.0 International license (CC-BY 3.0).

## References

[1] Latent tuberculosis infection: updated and consolidated guidelines for programmatic management.Geneva: World Health Organization; Licence: CC BY-NC-SA 3.0 IGO.2018; [cited 2019 Mar 6]. Reference Source 30277688

[2] Riley RL Mills CC Nyka W : Aerial Dissemination Of Pulmonary Tuberculosis A Two-Year Study Of Contagion In A Tuberculosis Ward. *Am J Epidemiol.* 1959;70(2):185–96. 10.1093/oxfordjournals.aje.a120069 7785671

[3] Vynnycky E Fine PE : The natural history of tuberculosis: the implications of age-dependent risks of disease and the role of reinfection. *Epidemiol Infect.* 1997;119(2):183–201. 10.1017/s0950268897007917 9363017 PMC2808840

[4] Pai M Denkinger CM Kik SV : Gamma Interferon Release Assays for Detection of *Mycobacterium tuberculosis* Infection. *Clin Microbiol Rev.* 2014;27(1):3–20. 10.1128/CMR.00034-13 24396134 PMC3910908

[5] Theel ES Hilgart H Breen-Lyles M : Comparison of the QuantiFERON-TB Gold Plus and QuantiFERON-TB Gold In-Tube Interferon Gamma Release Assays in Patients at Risk for Tuberculosis and in Health Care Workers. *J Clin Microbiol.* 2018;56(7):e00614–18. 10.1128/JCM.00614-18 29743310 PMC6018330

[6] Tsiouris SJ Coetzee D Toro PL : Sensitivity Analysis and Potential Uses of a Novel Gamma Interferon Release Assay for Diagnosis of Tuberculosis. *J Clin Microbiol.* 2006;44(8):2844–50. 10.1128/JCM.02411-05 16891501 PMC1594653

[7] Andersen P Munk ME Pollock JM : Specific immune-based diagnosis of tuberculosis. *Lancet.* 2000;356(9235):1099–104. 10.1016/s0140-6736(00)02742-2 11009160

[8] Yi L Sasaki Y Nagai H : Evaluation of QuantiFERON-TB Gold Plus for Detection of *Mycobacterium tuberculosis* infection in Japan. *Sci Rep.* 2016;6:30617. 10.1038/srep30617 27470684 PMC4965764

[9] Metcalfe JZ Cattamanchi A McCulloch CE : Test Variability of the QuantiFERON-TB Gold In-Tube Assay in Clinical Practice. *Am J Respir Crit Care Med.* 2013;187(2):206–11. 10.1164/rccm.201203-0430OC 23103734 PMC3570654

[10] Fielding K : Data set for the comparison of the performance of QuantiFERON-TB Gold Plus with QuantiFERON-T Gold in-tube among highly TB exposed gold miners in South Africa.[Data Collection]. London School of Hygiene & Tropical Medicine, London, United Kingdom.2020. 10.17037/DATA.00001891

[11] Telisinghe L Amofa-Sekyi M Maluzi K : The sensitivity of the QuantiFERON ^®^-TB Gold Plus assay in Zambian adults with active tuberculosis. *Int J Tuberc Lung Dis.* 2017;21(6):690–6. 10.5588/ijtld.16.0764 28482964 PMC5424670

[12] Moon HW Gaur RL Tien SSH : Evaluation of QuantiFERON-TB Gold-Plus in Health Care Workers in a Low-Incidence Setting. *J Clin Microbiol.* 2017;55(6):1650–7. 10.1128/JCM.02498-16 28298455 PMC5442521

[13] Hoffmann H Avsar K Göres R : Equal sensitivity of the new generation QuantiFERON-TB Gold *plus* in direct comparison with the previous test version QuantiFERON-TB Gold IT. *Clin Microbiol Infect.* 2016;22(8):701–3. 10.1016/j.cmi.2016.05.006 27184875

[14] Barcellini L Borroni E Brown J : First evaluation of QuantiFERON-TB Gold Plus performance in contact screening. *Eur Respir J.* 2016;48(5):1411–9. 10.1183/13993003.00510-2016 27390280

[15] Petruccioli E Vanini V Chiacchio T : Analytical evaluation of QuantiFERON- Plus and QuantiFERON- Gold In-tube assays in subjects with or without tuberculosis. *Tuberculosis (Edinb).* 2017;106:38–43. 10.1016/j.tube.2017.06.002 28802403

[16] Shao L Zhang W Zhang S : Potent immune responses of Ag-specific Vgamma2Vdelta2+ T cells and CD8+ T cells associated with latent stage of Mycobacterium tuberculosis coinfection in HIV-1-infected humans. *AIDS.* 2008;22(17):2241–50. 10.1097/QAD.0b013e3283117f18 18981763 PMC2743094

[17] Rose MV Kimaro G Nissen TN : QuantiFERON ^®^-TB Gold In-Tube Performance for Diagnosing Active Tuberculosis in Children and Adults in a High Burden Setting. *PLoS One.* 2012;7(7):e37851. 10.1371/journal.pone.0037851 22808002 PMC3395691

[18] Santin M Muñoz L Rigau D : Interferon-γ Release Assays for the Diagnosis of Tuberculosis and Tuberculosis Infection in HIV-Infected Adults: A Systematic Review and Meta-Analysis. *PLoS One.* 2012;7(3):e32482. 10.1371/journal.pone.0032482 22403663 PMC3293815

[19] Whitworth WC Hamilton LR Goodwin DJ : Within-Subject Interlaboratory Variability of QuantiFERON-TB Gold In-Tube Tests. *PLoS One.* 2012;7(9):e43790. 10.1371/journal.pone.0043790 22970142 PMC3435391

[20] Detjen AK Loebenberg L Grewal HMS : Short-Term Reproducibility of a Commercial Interferon Gamma Release Assay. *Clin Vaccine Immunol.* 2009;16(8):1170–5. 10.1128/CVI.00168-09 19535542 PMC2725540

[21] Banaei N Gaur RL Pai M : Interferon Gamma Release Assays for Latent Tuberculosis: What Are the Sources of Variability? *J Clin Microbiol.* 2016;54(4):845–50. 10.1128/JCM.02803-15 26763969 PMC4809912

[22] Rozot V Vigano S Mazza‐Stalder J : *Mycobacterium tuberculosis*-specific CD8 ^+^ T cells are functionally and phenotypically different between latent infection and active disease. *Eur J Immunol.* 2013;43(6):1568–77. 10.1002/eji.201243262 23456989 PMC6535091

[23] Day CL Abrahams DA Lerumo L : Functional capacity of *Mycobacterium tuberculosis*-specific T cell responses in humans is associated with mycobacterial load. *J Immunol.* 2011;187(5):2222–32. 10.4049/jimmunol.1101122 21775682 PMC3159795

[24] Lewinsohn DA Heinzel AS Gardner JM : *Mycobacterium tuberculosis*–specific CD8 ^+^ T Cells Preferentially Recognize Heavily Infected Cells. *Am J Respir Crit Care Med.* 2003;168(11):1346–52. 10.1164/rccm.200306-837OC 12969871

[25] Chiacchio T Petruccioli E Vanini V : Polyfunctional T-cells and effector memory phenotype are associated with active TB in HIV-infected patients. *J Infect.* 2014;69(6):533–45. 10.1016/j.jinf.2014.06.009 24975174

[26] Lancioni C Nyendak M Kiguli S : CD8 ^+^ T Cells Provide an Immunologic Signature of Tuberculosis in Young Children. *Am J Respir Crit Care Med.* 2012;185(2):206–12. 10.1164/rccm.201107-1355OC 22071329 PMC3297089

[27] Barcellini L Borroni E Brown J : First independent evaluation of QuantiFERON-TB Plus performance. *Eur Respir J.* 2016;47(5):1587–90. 10.1183/13993003.02033-2015 26869677

