# Supplementary material for: Comparing QuantiFERON-TB Gold Plus with QuantiFERON-TB Gold in-tube for diagnosis of latent tuberculosis infection among highly TB exposed gold miners in South Africa
Source: Gates Open Res. 2022 Aug 18;5:66. Originally published 2021 Mar 11. [Version 3] doi: 10.12688/gatesopenres.13191.3 (PMC10407057; doi:10.12688/gatesopenres.13191.3)
Supplement: Peer review history

# Peer review: Reviewer response for version 3

**DOI:** 10.21956/gatesopenres.15091.r34041

**Published:** 2023-08-07

**Authors:** Sonali Sarkar

**Affiliations:** 1 JIPMER, Puducherry, India

The authors have compared the agreement between QuantiFERON-TB Gold Plus and QuantiFERON-TB Gold In-Tube Interferon Gamma Release Assays in South African gold miners with the premise that the results might vary from the multiple studies done in low burden settings.

 The assumption that the agreement between the two tests can vary is a valid given the knowledge that the spectrum of the condition under study has some influence on sensitivity and specificity of the tests performed. Having said that, it is also noted that the artifactual variability that is introduced by excluding participants in whom the TB infection is difficult to diagnose, such as those with body mass index (BMI) <18.5, having serious medical conditions, HIV positive and current treatment for cancer, treatment with steroid tablets, inhalers or injections, overestimates the accuracy of the tests.(1) There are multiple studies reporting the agreement between the two tests in lower incidence setting. Results from repetition of a similar study in a high-burden setting was expected and does not contribute to the clinical decision of using the QFT-Plus in similar or other settings. The inputs required for justification for the use of QFT-Plus would come from directional hypothesis with minimal acceptable criteria set in calculation of sample size (2). An attempt at sample size calculation should have been done to justify the validity of the results.

 Minor comments regarding the study are as follows.

1. The flowchart of the number of participants does not match with the numbers stated in the text.
2. Typographical error in line 3 of the results section under the demographic characteristics, work-face to be corrected as work-force.

References

1. Leeflang MM, Rutjes AW, Reitsma JB, Hooft L, Bossuyt PM. Variation of a test's sensitivity and specificity with disease prevalence. CMAJ. 2013 Aug 6;185(11):E537-44. doi: 10.1503/cmaj.121286. Epub 2013 Jun 24. PMID: 23798453; PMCID: PMC3735771.
2. Korevaar, D.A., Gopalakrishna, G., Cohen, J.F. *et al.* Targeted test evaluation: a framework for designing diagnostic accuracy studies with clear study hypotheses. *Diagn Progn Res* **3**, 22 (2019). https://doi.org/10.1186/s41512-019-0069-2

Is the work clearly and accurately presented and does it cite the current literature?

Yes

If applicable, is the statistical analysis and its interpretation appropriate?

Yes

Are all the source data underlying the results available to ensure full reproducibility?

No

Is the study design appropriate and is the work technically sound?

Partly

Are the conclusions drawn adequately supported by the results?

Yes

Are sufficient details of methods and analysis provided to allow replication by others?

Yes

Reviewer Expertise:

Epidemiology of Tuberculosis

I confirm that I have read this submission and believe that I have an appropriate level of expertise to confirm that it is of an acceptable scientific standard.

## References

[1] : Variation of a test's sensitivity and specificity with disease prevalence. *CMAJ* .2013;185(11) : 10.1503/cmaj.121286 E537-44 10.1503/cmaj.121286 23798453PMC3735771

[2] : Targeted test evaluation: a framework for designing diagnostic accuracy studies with clear study hypotheses. *Diagn Progn Res* .2019;3: 10.1186/s41512-019-0069-2 22 10.1186/s41512-019-0069-2 31890896PMC6921417

# Peer review: Reviewer response for version 3

**DOI:** 10.21956/gatesopenres.15091.r34036

**Published:** 2023-08-02

**Authors:** Lucia Barcellini

**Affiliations:** 1 University of Milan, Milan, Italy

The study compared the performance of QuantiFERON-TB Gold-Plus (QFT-Plus) with the standard QFT-GIT assay in diagnosing latent tuberculosis infection in goldminers. QFT-Plus showed high agreement (94.7%) with QFT-GIT, suggesting similar performance. 

 The microbiological procedure used in the study is reliable, and the inclusion of subclinical TB testing with a culture is a strong aspect.

 However, the inclusion criteria have led to a significant reduction in the population included, and they are not entirely clear.

 While some criteria are clear, such as excluding previous TB patients or those currently on prophylaxis, the rationale behind others is less transparent.

 If the objective was to assess the agreement between the two tests, having a more diverse sample, including different age groups, individuals with low BMI, and those with comorbidities, could actually be advantageous.

 Please, clarify the specific rationale behind the chosen criteria and /or discuss them as a limitation.

Is the work clearly and accurately presented and does it cite the current literature?

Yes

If applicable, is the statistical analysis and its interpretation appropriate?

Yes

Are all the source data underlying the results available to ensure full reproducibility?

Yes

Is the study design appropriate and is the work technically sound?

Partly

Are the conclusions drawn adequately supported by the results?

Yes

Are sufficient details of methods and analysis provided to allow replication by others?

Yes

Reviewer Expertise:

Microbiology: diagnostic test for TB

I confirm that I have read this submission and believe that I have an appropriate level of expertise to confirm that it is of an acceptable scientific standard, however I have significant reservations, as outlined above.

# Peer review: Reviewer response for version 3

**DOI:** 10.21956/gatesopenres.15091.r32392

**Published:** 2022-08-25

**Authors:** Mark Hatherill

**Affiliations:** 1 South African Tuberculosis Vaccine Initiative (SATVI), University of Cape Town, Cape Town, South Africa

This revision is satisfactory, I have no further comments.

Is the work clearly and accurately presented and does it cite the current literature?

Yes

If applicable, is the statistical analysis and its interpretation appropriate?

Yes

Are all the source data underlying the results available to ensure full reproducibility?

No source data required

Is the study design appropriate and is the work technically sound?

Partly

Are the conclusions drawn adequately supported by the results?

Yes

Are sufficient details of methods and analysis provided to allow replication by others?

Yes

Reviewer Expertise:

NA

I confirm that I have read this submission and believe that I have an appropriate level of expertise to confirm that it is of an acceptable scientific standard.

# Peer review: Reviewer response for version 2

**DOI:** 10.21956/gatesopenres.14885.r31933

**Published:** 2022-06-06

**Authors:** Mark Hatherill

**Affiliations:** 1 South African Tuberculosis Vaccine Initiative (SATVI), University of Cape Town, Cape Town, South Africa

The authors have revised statements that “ *QFT-Plus showed similar performance to QFT-GIT*” to state that QFT-Plus showed high agreement with QFT-GIT, suggesting similar performance, since performance of these tests to differentiate true Mtb infection was not measured.

 However, in version 2 the abstract, introduction and methods still state incorrectly that the study compared performance of QFT-Plus with QFT-GIT. This needs to be corrected to reflect that agreement was tested, not performance.

Is the work clearly and accurately presented and does it cite the current literature?

Yes

If applicable, is the statistical analysis and its interpretation appropriate?

Yes

Are all the source data underlying the results available to ensure full reproducibility?

No source data required

Is the study design appropriate and is the work technically sound?

Partly

Are the conclusions drawn adequately supported by the results?

Yes

Are sufficient details of methods and analysis provided to allow replication by others?

Yes

Reviewer Expertise:

tuberculosis vaccinology

I confirm that I have read this submission and believe that I have an appropriate level of expertise to confirm that it is of an acceptable scientific standard, however I have significant reservations, as outlined above.

# Peer review: Reviewer response for version 1

**DOI:** 10.21956/gatesopenres.14396.r30700

**Published:** 2021-06-04

**Authors:** Mark Hatherill

**Affiliations:** 1 South African Tuberculosis Vaccine Initiative (SATVI), University of Cape Town, Cape Town, South Africa

Ntshiqa *et al. *report a comparison of QuantiFERON-TB Gold Plus with QuantiFERON-TB Gold in-tube among highly TB exposed gold miners, showing that agreement was high (94%). 

**Major comments:**

- The data analysed are from a subset of a larger parent study and the 304 participants available for direct comparison form only 1.8% of the 17,030 who consented to screening; and the study participants included in the analysis are all very highly exposed to Mtb over a long period. i.e. It is not clear if the study population is representative of other settings, or to what extent the results are generalisable.

**Minor comments:**

- Abstract: Typo 33 years of age?
- Study limitations: The statement that “it is unlikely that the LTBI status would change over a relatively short period of follow-up” does not justify the lack of confirmatory testing.
- Discussion: No TST information is included in the title, abstract or discussion, but is included in the methods and main results. Suggest TST information is either included in the title and abstract, and concordance/discordance with the IGRA tests is interpreted and discussed further, or TST is removed entirely from this manuscript.
- The observation that TB2-TB1 was associated with BMI deserves further discussion.
- Conclusion (abstract and main text): Here and elsewhere - suggest statements that “QFT-Plus showed similar performance to QFT-GIT” should be revised to state that QFT-Plus showed high agreement with QFT-GIT, or qualified as per the Discussion “suggesting similar performance” since performance of these tests to differentiate true Mtb infection was not measured.

Is the work clearly and accurately presented and does it cite the current literature?

Yes

If applicable, is the statistical analysis and its interpretation appropriate?

Yes

Are all the source data underlying the results available to ensure full reproducibility?

No source data required

Is the study design appropriate and is the work technically sound?

Partly

Are the conclusions drawn adequately supported by the results?

Yes

Are sufficient details of methods and analysis provided to allow replication by others?

Yes

Reviewer Expertise:

tuberculosis vaccinology

I confirm that I have read this submission and believe that I have an appropriate level of expertise to confirm that it is of an acceptable scientific standard, however I have significant reservations, as outlined above.
